# Effects of non-pharmacological interventions for preventing delirium in general ward inpatients: A systematic review & meta-analysis of randomized controlled trials

**DOI:** 10.1371/journal.pone.0268024

**Published:** 2022-05-06

**Authors:** Yun Hee Kim, Nam Young Kim, Seang Ryu

**Affiliations:** 1 Department of Nursing, Mokpo National University, Muan, Korea; 2 Department of Nursing, Jungwon University, Goesan, Korea; University of Rome ’La Sapienza’, ITALY

## Abstract

The purpose of this study was to identify the types and contents of non-pharmacological delirium prevention interventions applied to inpatients in general wards, and to verified the effectiveness of the interventions on the incidence of delirium. We performed an extensive search of bibliographic databases and registries (CENTRAL, MEDLINE, EMBASE, CINAHL, ClinicalTrials.gov and WHO International Clinical Trials Registry Platform, PubMed and Google Scholar, and Korean DB such as RISS, DBpia, KISS, NDSL and KCI) using terms to identify delirium, prevention, and non-pharmacological. We searched all databases from their inception to January 2021 and imposed restriction on language of publication in English and Korean. We included studies if they were conducted as all types of randomized controlled trials (RCT), involving adult patients aged 19 years or more who were admitted to a general ward. We included trials comparing non-pharmacological intervention versus usual care. The entire process of data selection and extraction, assessment of risk of bias with ROB2.O was independently performed by three researchers. The estimated effect size was an odds ratio (OR) and 95% confidence interval. The fixed effects model and general inverse variance estimation method were adopted. The type of non-pharmacological delirium prevention interventions for inpatients in general ward was mainly multi-component intervention to correct delirium risk factors. The content and intensity of non-pharmacological interventions varied greatly depending on the characteristics of the patient and the clinical situation. As a result of the meta-analysis, non-pharmacological multi-component intervention was effective in reducing the incidence of delirium, and it was confirmed that it was effective in reducing the incidence of delirium in both the internal and surgical wards. It was confirmed by quantitative evidence that non-pharmacological interventions, especially multi-component interventions, were effective in preventing delirium in general ward inpatients.

## Introduction

Delirium is an acute clinical syndrome showing decreases of consciousness and concentration with cognitive and perceptual disturbances [1]. It is the second most common syndrome after depression in general ward inpatients. It occurs in about 14~55% of general ward inpatients [2]. Delirium increase the length of hospital day by about 1.8 times, discharge to long-term care facilities by about 1.32 times [3], and the mortality by about 9% [4], and aggravate the cognitive and functional decline [3, 5]. In addition, medical cost to be paid due to the occurrence of delirium is more than twice that for patients without delirium [6]. As such, it is very important to prevent delirium in general ward inpatients because the occurrence of delirium poses economic and psychological burden not only to the patient, but also to the family and medical institutions taking care of the patient.

Delirium is caused by cognitive impairment, sleep deprivation, immobility, vision impairment, hearing impairment, dehydration, and other risk factors [7]. It has been reported that when a patient has three or more risk factors, the delirium incidence rate is about 30%~100% [8]. When delirium occurs, it is difficult to reduce its severity or duration. However, delirium is potentially preventable in 30~40% of patients who develop it [8]. Therefore, it is necessary to evaluate risk factors of delirium at an early stage and develop an active strategy to prevent it.

Pharmacologic intervention has been widely applied to prevent delirium. However, previous systematic reviews have shown that a pharmacologic intervention is not effective in reducing the incidence of delirium [5, 9]. On the other hand, drugs may induce insomnia, nausea, vomiting, drowsiness, and headache that can aggravate delirium symptoms with various side effects [10, 11]. It has been reported that drug interventions are not effective in preventing delirium [12]. Thus, non-pharmacological preventive interventions are attracting attention.

Non-pharmacological interventions can be divided into single component interventions and multicomponent interventions aimed at resolving modifiable risk factors among risk factors for delirium. Music therapy and staff education are single interventions for general ward inpatients [13]. Although delirium can be caused by a single factor, it is known that it is caused by various risk factors [14]. Thus, multi-component intervention consisting of at least two factors is recommended in the clinical field to solve modifiable risk factors for the occurrence of delirium [2]. The Hospitalized Elderly Life Program (HELP) proposed to correct six risk factors (cognitive impairment, sleep deprivation, immobility, vision impairment, hearing impairment, dehydration) [7] is a representative example. Based on HELP, the National Institute for Health and Care Excellence (NICE) guideline [15] has assessed risk factors for delirium at the time a patient is hospitalized. Based on the assessment results, NICE guideline recommended multicomponent intervention consisting of 10 contents, including disorientation. The Scottish Intercollegiate Guidelines Network (SIGN) guideline [16] recommends reducing or modifying the risk factors for delirium such as preventing dehydration and hypoxia, maintaining sleep, reducing psychological stress through communication and environmental management, and sensory impairment. Pharmacological multi-component intervention is recommended to prevent delirium. These guidelines reflect the most recent knowledge among the delirium prevention and intervention guidelines published so far [17]. The present study intends to use intervention contents presented in the NICE guideline [15] and the SIGN guideline [16] as framework for a systematic review. As mentioned earlier, non-pharmacological delirium prevention intervention guidelines recommend multicomponent intervention. However, single component intervention is still being applied in the clinical field according to the characteristics and environment of the subject [13].

There are various types of non-pharmacological interventions for delirium prevention. Most of previous systematic reviews and meta-analysis studies on non-pharmacological interventions for inpatient delirium prevention have focused on multicomponent intervention [9, 18, 19]. However, prior systematic review studies [9, 18, 19] have included cancer patients and critically ill patients admitted to the ward. In addition, clinical characteristics of patients were different from those admitted to the general ward. In particular, in the case of cancer patients, the location of the disease, which is a risk factor that cannot be modified for commonly known risk factors for delirium, has been reported as a factor affecting the occurrence of delirium [20]. Therefore, there is a limit to using results of previous studies [9, 18, 19] as a scientific basis to provide non-pharmacological delirium prevention or intervention for inpatients. In addition, systematic review studies on non-pharmacologic single component interventions are not active [13]. They show limitations in presenting a comprehensive scientific basis for non-pharmacological delirium prevention interventions in general ward inpatients.

The purpose of this study was to identify the types and contents of non-pharmacological delirium prevention interventions applied to inpatients in general wards, and to verified the effectiveness of non-pharmacological delirium prevention interventions on the incidence of delirium. That would provide a scientific evidence for non-pharmacological delirium prevention interventions for general ward inpatients.

## Materials and methods

### Eligibility criteria

In this study, core questions (population, intervention, comparison, outcome [PICO]) were selected according to the Cochrane Handbook for Systematic Reviews of Interventions [21]. Specific PICO was as follows: (1) Population: adult patients aged 19 years or more who were admitted to a general ward; (2) Intervention: non-pharmacological intervention for the prevention of delirium. There were no restrictions on the type of intervention or the number of interventions provided; (3) Comparison: c provided for the prevention of delirium in general ward inpatients, (4) Outcome: the delirium incidence rate measured after non-pharmacological delirium prevention intervention was applied to general ward inpatients, and (5) Study design: Randomized Clinical Trial (RCT), cluster RCT, and step wedge RCT were included.

Among key questions in the data selection criteria, population, intervention, and comparison were included. To systematically review studies on the effectiveness of delirium prevention intervention, outcome variables for delirium prevention intervention were not included. Non-experimental studies such as studies on critically ill patients, studies on cancer patients admitted to hospital wards, studies on patients admitted to nursing hospitals, studies that applied drug intervention to prevent delirium, observational studies, and review studies were also excluded from data selection criteria. This study did not apply for IRB approval because only literature was used as a research subjects.

### Search strategy

One researcher with extensive experience in systematic literature review and meta-analysis research conducted a systematic data search on January 11 to 15, 2021. The database was selected based on the COSI (COre, Standard, Ideal) model presented by the National Library of Medicine in the United States. As a search source, core databases such as Ovid-MEDLINE, EMBASE, and CENTRAL (Cochrane Central Register of Controlled Trials) were used. The standard database CINAHL was also used. An ideal database was added by manual search using the snowball method. PubMed and Google Scholar were also used. Search engines in Korea, including Research Information Sharing Service (RISS), Database Periodical Information Academic (DBpia), Korean Studies Information Service System (KISS), National Assembly Library (National Assembly Library), the National Digital Science Library (NDSL), and the Korea Citation Index (KCI) were used. Based on ‘Medical Subject Headings, MeSH’ and ‘EMTREE’, search terms were selected and search strategies were established. Search functions such as Boolean operators and truncated search were used. ‘Delirium’ [MeSH Terms], ‘(deliri* OR acute confusion* OR acute organic psychosyndrome OR acute brain syndrome OR metabolic encephalopathy OR acute psycho-organic syndrome OR clouded state OR clouding of consciousness OR exogenous psychosis OR toxic psychosis OR toxic confusion OR obnubilat*)’ [Textword], Interventionk ‘Primary Prevention’ [MeSH Terms], ‘(prevent* OR reduc* OR stop* OR taper* OR cut* down" OR avoid*)’[Textword] were used to select participants. The study design used the HSSS search filter suggested by Cochrane. To supplement the search, a handwritten search based on references was added, and there was no restriction on the year of publication.

### Data selection and collection

Searched data were managed using the bibliographic management program (EndNote v20.0). First, duplicate literature was removed. The primary selection was based on the title and abstract of the paper. According to data selection and exclusion criteria, the original text of the paper was reviewed. The literature to be included in the systematic review was finally selected through secondary selection.

Data were extracted using the data collection format designed by this research team. Contents included in literature characteristics included author, year, country name, number of participants and dropout rate, age, gender, type and contents of intervention, dose, delivery method, and so on. Outcome variable included the incidence of delirium.

The first screening of data selection was performed on January 18–22, 2021. The second screening was performed on January 25-February 12, 2021. Quality assessment was performed on February 15–19, 2021. Collection of data was performed on March 1–30, 2021. The entire process of data selection, quality evaluation, and data collection was independently performed by three researchers. In case of disagreement, results were reviewed and discussed based on the selection and exclusion criteria to derive a consistent result.

### Risk of bias assessment

For assessment of risk of bias, the Cochrane Coalition’s RCT quality evaluation tool (Risk of Bias 2.0, RoB 2.0) [22] was used. RoB 2.0 is divided into five areas (’Risk of bias arising from the randomization process’, ’Risk of bias due to deviations from the intended interventions’, ’Risk of bias due to missing outcome data’, ’Risk of bias in the measurement of the outcome’, and ’Risk of bias in a selection of the reported result’). The signal questions were evaluated first, After that, ’having a risk of bias’ was evaluated as ‘yes’, ‘probably yes’, ‘no’, ‘probably no’, or ‘no information’. Next, the final decision for each area was evaluated as low risk of bias, high risk, and some concerns according to the evaluation algorithm presented in RoB 2.0 using the evaluation result of the signal question for each area. Finally, according to the overall literature quality evaluation criteria presented in RoB 2.0, the final judgment of the risk of bias was made with low, high, and some concerns [21]. Quality evaluation was conducted independently by three researchers after securing the original text. In case of disagreement, consensus results were obtained through discussion.

### Data analysis

To calculate the effect size on delirium incidence rate, the Cochrane Coalition Review Manager version 5.4 was used for analysis. Since the delirium incidence rate was dichotomous, meta-analysis was performed by calculating the number of delirium occurrence frequencies in the experimental group and the control group. The effect estimate was described with an odds ratio (OR) and 95% confidence interval. As for the statistical model, fixed effects model is recommended when I^2^ is 50% or less [23]. In this study, fixed effects model and general inverse variance estimation method were used.

The heterogeneity between the studies was evaluated using the Chi-square test and Higgin’s I^2^ statistics after confirming whether the direction of the effect size and the confidence interval overlapped with each other through a forest plot, a visual method. I^2^ was interpreted as small size heterogeneity if I^2^ ≤ 25%, moderate heterogeneity if 25% < I^2^ ≤ 75%, and large heterogeneity if I^2^ > 75% or more [21].

Publication bias of the literature was visually confirmed through funnel plots. If funnel plots were asymmetrical, data could be biased. Thus, Egger’s test was performed. For the degree of influence of publication bias, the effect size was estimated by filling in the estimated effect size of studies. The trim and fill method was used to compare and analyze values before and after correction to confirm the degree of influence of publication bias [24].

During quantitative synthesis, results were not presented in Part 1 [A11 in S1 Appendix] of the multicomponent intervention study. A request for results was sent to the author by e-mail. However, there was no reply. Thus, they were excluded from meta-analysis. In the case of single component intervention, the content of the intervention was different. Therefore, it was not used for quantitative synthesis. It was only used for qualitative synthesis.

## Results

### Search and selection

**Fig 1:**
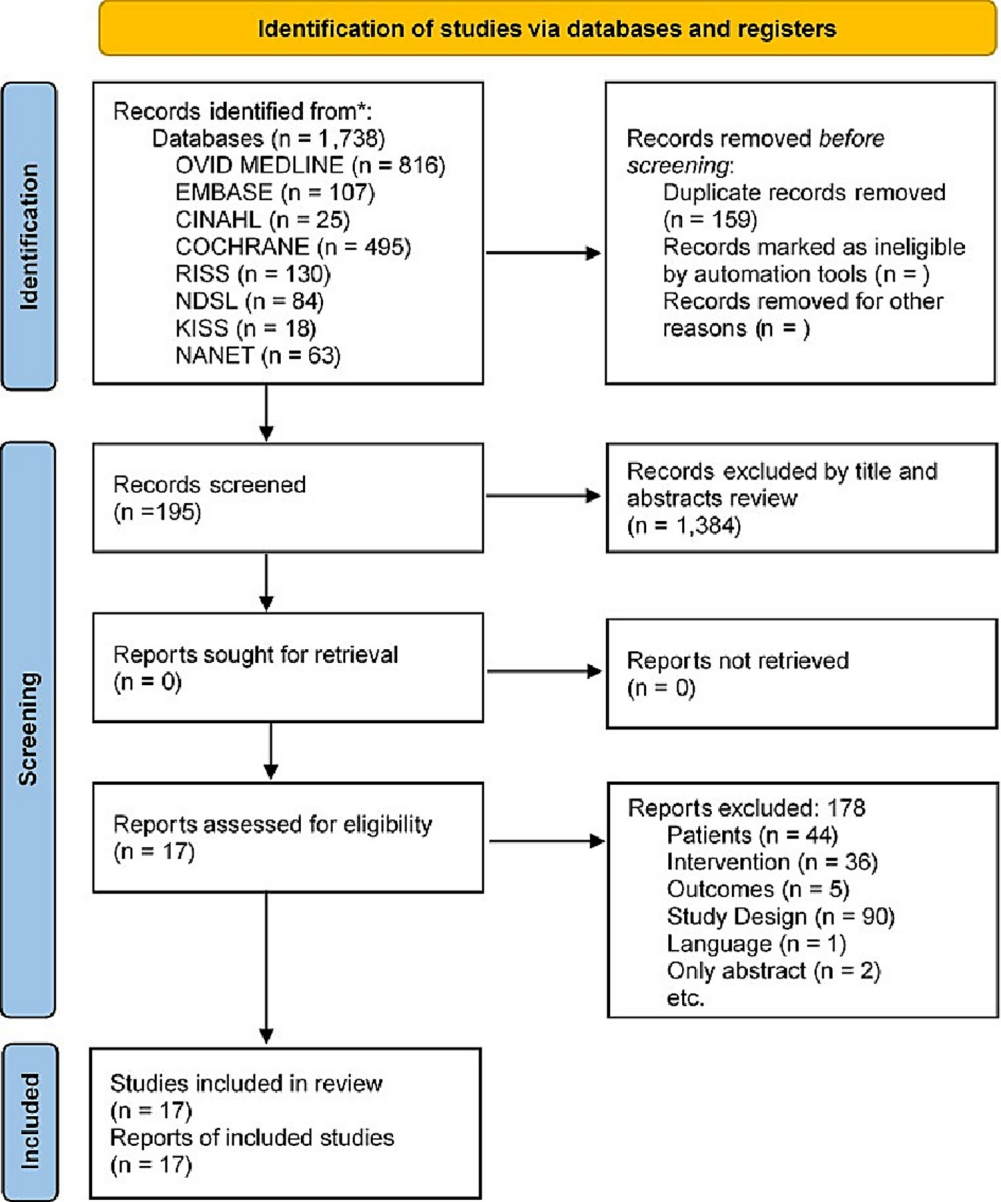
PRISMA flow.

A total of 1,738 studies were collected through a systematic literature search process. After removing 159 duplicate studies, 1,384 were additionally removed based on the title and abstract. After reviewing original texts of 195 selected studies, a total of 178 were excluded: 44 that did not meet the subject criteria, 36 that did not meet the non-pharmacological preventive intervention, five that did not meet the outcome variable criteria, 90 that did not design RCTs, two that lacked information on outcome variables and interventions in the abstract, and one that was not English or Korean. Finally, 17 papers were included in the systematic literature review and meta-analysis (Fig 1).

### Risk of bias

**Fig 2:**
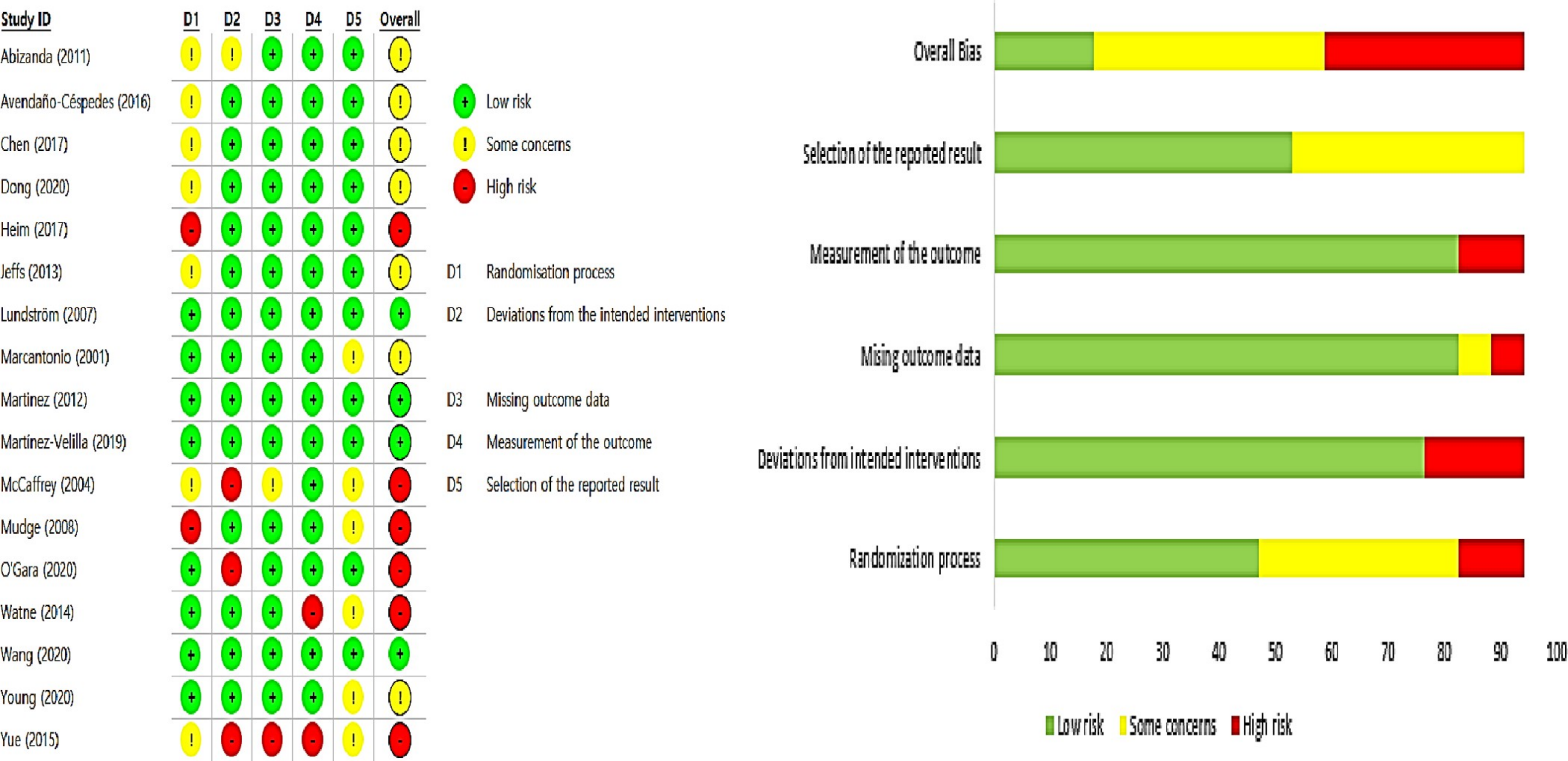
Risk of bias.

Looking at the risk of bias assessment of the literature to be analyzed, the risk of bias occurring during the randomization process was low in 8 cases, the risk of bias due to deviation from the intended intervention was low in 14 cases, the risk of bias due to missing intervention outcome data was low in 15 trials, the risk of bias in the intervention outcome measurement was low in 15 trials, and the reported risk of selection bias was low in 11 trials. As a result of the final determination according to ROB 2.0 evaluation algorithm, four studies with low risk of bias, seven studies with some concern, and six studies with a high risk of bias were found (Fig 2).

### Study characteristics

All 17 papers to be analyzed were published between 2001 and 2020, of which 16 were published in academic journals and one was an abstract. Regarding the place where the study was conducted, eight were surgical wards, six were internal medicine wards, and five were geriatric wards. There were total of 4428 subjects (2353 in the experimental group and 2221 in the control group), of which 1011 in the experimental group and 902 in the control group were males. The dropout rate was 4.21% in the experimental group and 2.12% in the control group. The age distribution of subjects was 70–87.6 years in the experimental group and 69–87.0 years in the control group. Regarding the type of non-pharmacological delirium prevention intervention, there were three single-component interventions: exercise programs, music listening, and cognitive training. Fourteen multicomponent interventions were identified, modified HELP was applied in three studies and cognitive training was implemented in two studies. Ten multicomponent interventions were effective in reducing the incidence of delirium, and three of the ten multicomponent interventions used mHELP. In seven studies that reported the number of delirium prevention interventions, it varied from 1 to 5 times/day, while 11 studies did not report it. The total duration of delirium prevention intervention was from discharge to discharge in six studies, 5–7 days in one study, and no report in 10 studies. In ten studies, nurses participated as mediators. Geriatrician, geriatric psychiatrist, patient family, patient, exercise specialist, trained volunteer, physical therapist, occupational therapist, nutritionist, interdisciplinary team, psychology student, and certified allied health assistant participated as an arbitrator. Comparative intervention was routine care in 14 studies. As a tool for diagnosing delirium, CAM was used in 14 studies. OBS, numerical score and patient chart also were used in one study, respectively (Table 1).

**Table 1:** Descriptive summary of included studies (N = 17).

Studies		Patients		Interventions			Comparison	Outcomes
Author (Year) Country	Setting	SS/M/DR n (%)	Age M (SD) or Median (IQR)					Incidence of Delirium n (%)
				Content (Type)	Frequency/Duration	Provider		
Abizanda et al (2011) Spain	AGU	I:198/86/25(12.6) C:202/87/34(16.8)	I: 83.3 (6.5) C: 83.7 (6.1)	Occupational therapy intervention (M)	5 times a week/until discharge	Occupational therapist	CTM	I: 27 (15.6) C: 39 (23.2) (*p*>0.05) MT: CAM
Avendaño-Céspedes et al (2016) Spain	AGU	I:21/10/0 (0) C: 29/16/0 (0)	I: 85.8(6.2) C: 87.0(4.9)	Preventive multicomponent nurse led intervention (M)	Once a day/Until discharge	Nurse	UC	I: 3 (14.3) C: 12 (41.4) (*p* = .039) MT: CAM
Chen et al. (2017) Taiwan	GI	I: 197/111/0 (0) C: 180/108/0 (0)	I: 74.3(5.8) C: 74.8 (6.0)	mHELP (M)	Once a day/Until discharge	mHELP nurse	UC	I: 13 (6.6) C: 27 (15.0) (*p* = .008) MT: CAM
Dong et al. (2020) China	University hospital	I: 53/32/3 (5.6) C: 53/34/0 (0)	I: 75.87 (4.32) C: 76.23 (4.58)	mHELP (M)	NR/2 weeks or if discharged within 14 days, until discharged	Nurse	UC	I: 2 (4.0) C: 9 (17.0) (*p* = .033) MT: CAM
Heim et al. (2017) Netherlands	Geriatrics, Cardiology Medical unit Surgical unit OS	I: 344/134/121 (35.2) C: 174/40/57 (32.7)	I: 82.5 (78–87) C:85 (80–88)	HELP (M)	NR/Until discharge	Bedside trained volunteer/Nurse practitioner	UC	I: 34 (15.2) C: 28 (23.9) (CI = 0.22–1.19) MT: CAM
Jeffs et al. (2013) Australia	Medical unit	I: 305/137/0(0) C: 344/171/1(0.3)	I: 79.6 (7.5) C:79.1 (7.9)	Enhanced exercise and cognitive program (M)	Twice (day)/NR	Certified allied health assistant	UC	I: 15 (4.9) C: 21 (6.1) (*p* = 0.6) MT: CAM
Lundström et al (2007) Sweden	OS	I: 102/28/0(0) C: 97/23/0(0)	I: 82.3 (6.6) C: 82.0 (5.6)	Multi-factorial intervention (M)	NR/NR	Nurse, LPN, geriatricians, occupational therapist, physiotherapist, dietician	UC	I: 56 (54.9) C: 73 (75.3) MT: OBS (*p* = .003)
Marcantonio et al. (2001) USA	OS	I: 62/13/0(0) C: 64/14/0(0.0)	I: 78 (8) C:80 (8)	Proactive geriatrics consultation (M)	5 times after first visit and 3 times after follow-up visit /NR	Geriatrician	UC	I: 20 (32) C: 32 (50) (*p* = .04) MT: CAM
Martinez (2012) Chile	Medical unit	I: 144/84/0(0.0) C: 143/96/0(0.0)	I: 78.1 (6.3) C:78.3 (6.1)	Prophylactic environmental management (M)	NR/NR	Family member	SM	I: 8 (5.6) C: 19 (13.3) (*p* = .027) MT: CAM
Martínez-Velilla et al. (2019) Spain	ACE unit	I: 185/85/0(0.0) C: 185/76/0(0.0)	I: 87.6 (4.6) C:87.1 (5.2)	Exercise program (S)	Twice (day)/5-7 days	Fitness specialist	UC	I: 15 (8.1) C: 27 (14.6) (*p* = .12) MT: CAM
McCaffrey et al. (2004) USA	OS	I: NR C: NR	I: NR C: NR	Music listening (S)	NR/NR	Nurse/Patients himself/Family member	SPC	I: NR C: NR (*p* = .001) MT: numerical score
Mudge et al. (2008) Australia	Medical unit	I: 62/27/0(0.0) C: 62/25/0(0.0)	I: 81.7 (7.8) C:82.4 (7.4)	Integrated approach (M)	NR/NR	Physiotherapist/Multidisciplinary team/psychology students	UC	I: 12 (19.4) C: 22 (35.5) (*p* = .04) MT: patient’s chart
O’Gara et al. (2020) USA	Surgical unit	I: 22/14/2(9.1) C: 23/15/3(13.0)	I: 70 (6) C:69 (7)	Cognitive training (S)	NR/NR	Investigator	UC	I: 5 (25.0) C: 3 (15.0) (*p* = .69) MT: CAM
Watne et al. (2014) Norway	AGU	I: 163/42/0 (0.0) C: 166/38/0 (0.0)	I: 84 (55–99) C:85(46–101)	Cognitive training (M)	NR/NR	Geriatrician/Nurse/Physiotherapist/Occupational therapist	UC	I: 80 (49.1) C: 86 (51.8) (*p* = .51) MT: CAM
Wang et al (2020) China	Surgical unit	I: 152/96/0 (0.0) C: 129/75/0 (0.0)	I:74.2(5.53) C:75.28(4.73)	Tailored-HELP (M)	NR/7 days or until discharge	Nurse, family	UC	I: 4 (2.6) C: 25 (19.4) (*p* < .001) MT:CAM
Young et al (2020) UK	OS	I: 343/112/0(0.0) C: 370/114/0(0.0)	I: 82.5 (7.9) C:83.0 (7.8)	Prevention of delirium (M)	NR/NR	Senior nurse Ward manager Voluntary -services manager	UC	I: 24 (7.0) C: 33 (8.9) (*p* = .22) MT: CAM
Yue (2015) ^†^ China	Elective surgery	I: 52/NR/NR C: 48/NR/NR	I: NR C: NR	mHELP (M)	NR/NR	Physicians/Nurse Physical therapist/ Nutritionists	UC	I: 8 (15.4) C: 17 (35.4) (*p* = .03) MT: CAM
	Total sample size		I: 2353 C: 2221					
	Mean DR (%)		I: 4.21 C: 2.12					

† Proceeding; I = Intervention; C = Control; SD = Study design; NR = Not reported; DR = Drop rate; M = Male; AGU = Acute geriatric unit; GI: Gastrointestinal; OS: Orthopedics surgery; ACE = Acute care of elderly; M = Multicomponent; S = Single; MT = Measurement Tool; CTM = Conventional treatment model; UC = Usual care; SM = Standard management; SPC = Standard postoperative care; CAM = Confusion assessment method; LPN = Licensed practical nurses; OBS = Organic brain syndrome scale.

In this study, based on non-pharmacological delirium prevention interventions recommended by the NICE guideline [15] and the SIGN guideline [16] for the reduction and resolution of risk factors for delirium, non-pharmacological delirium prevention intervention components described in the analysis target literature were systematically analyzed. First, mediation components commonly presented in NICE [15] and SIGN [16] guidelines were reviewed. Orientation was performed in 10 studies [A1-A6, A9, A15-A17 in S1 Appendix]. Hearing and visual aid was in five studies [A4, A5, A9, A15, A17 in S1 Appendix]. Sleep pattern and hygiene were in five studies [A4, A5, A7, A15, A17 in S1 Appendix]. Early mobilization was in 12 studies [A1, A3, A5-A8, A10, A12, A14-A17 in S1 Appendix]. Pain control was in eight studies [A2, A4, A7, A8, A14-A17 in S1 Appendix]. Optimal hydration was in seven studies [A2, A4, A5, A8, A15-A17 in S1 Appendix]. Nutrition was in nine studies [A2-A4, A7, A8, A14-A17 in S1 Appendix]. Five studies [A2, A4, A7, A8, A15 in S1 Appendix] described bladder and bowel function. Oxygen supply was used in six studies [A2, A4, A7, A8, A14, A17 in S1 Appendix]. Infection control was performed in five studies [A4, A8, A5, A16, A17 in S1 Appendix]. Four studies [A1, A2, A5, A7 in S1 Appendix] performed tailored multicomponent intervention only recommended by the NICE guideline [15]. Medication review was performed in five studies [A2, A4, A8, A14, A15 in S1 Appendix]. Two studies [A7, A8 in S1 Appendix] performed prevention, early identification, and treatment of postoperative complications. Four studies [A3, A6, A9, A15 in S1 Appendix] described other therapeutic activities only recommended by the SIGN guideline [16]. One study [A4 in S1 Appendix] performed cognitive therapy. One study [A12 in S1 Appendix] performed cognitive stimulation. One study [A11 in S1 Appendix] used music therapy. One study [A13 in S1 Appendix] used a mobile application featuring program. One study [A14 in S1 Appendix] performed comprehensive geriatric assessment (Table 2).

**Table 2:** Types of non-pharmacological delirium prevention interventions based on NICE and SIGN guideline (*N* = 15).

Studies	Abizanda et al (2011)	Avendaño-Céspedes et al (2016)	Chen et al. (2017)	Dong et al. (2020)	Heim et al. (2017)	Jeffs et al. (2013)	Lundstr-öm et al (2007)	Marcanto-nio et al. (2001)	Martinez et al. (2012)	Martínez-Velilla et al. (2019)	McCaffery et al. (2004)	Mudge et al. (2008)	O’Gara et al. (2020)	Watne et al.(2014)	Wang et al (2020)	Young et al. (2020)	Yue (2015)
Orientation	O	O	O	O	O	O			O						O	O	O
Hearing/visual aids				O	O				O						O		O
Sleep pattern/hygiene				O	O		O								O		O
Early mobilization	O		O		O	O	O	O		O		O		O	O	O	O
Pain control		O		O			O	O						O	O	O	O
Optimal hydration		O		O	O			O							O	O	O
Nutrition		O	O	O			O	O						O	O	O	O
Bladder/bowel function		O		O			O	O							O		
Oxygen supply		O		O			O	O						O			O
Infection control				O				O							O	O	O
^†^Prevention, early identification /treatment of postoperative complications							O	O									
^††^Tailored multi-component intervention	O	O					O								O		
^††^Medication review		O		O				O						O	O		
Etc.			Therapeutic activites	Cognitive therapy		Therapeutic activites			Therapeutic activites		Music	Cognitive stimulation	Mobile application featuring program	Comprehensive Geriatric assessment	Therapeutic activities		
Total	3	8	4	11	5	3	8	9	3	1	1	2	1	6	12	7	9

^†^ Only SIGN guideline^;^

^††^ only NICE.

### Effects of non-pharmacological interventions for preventing delirium

#### Overall effect size on incidence of delirium

**Fig 3:**
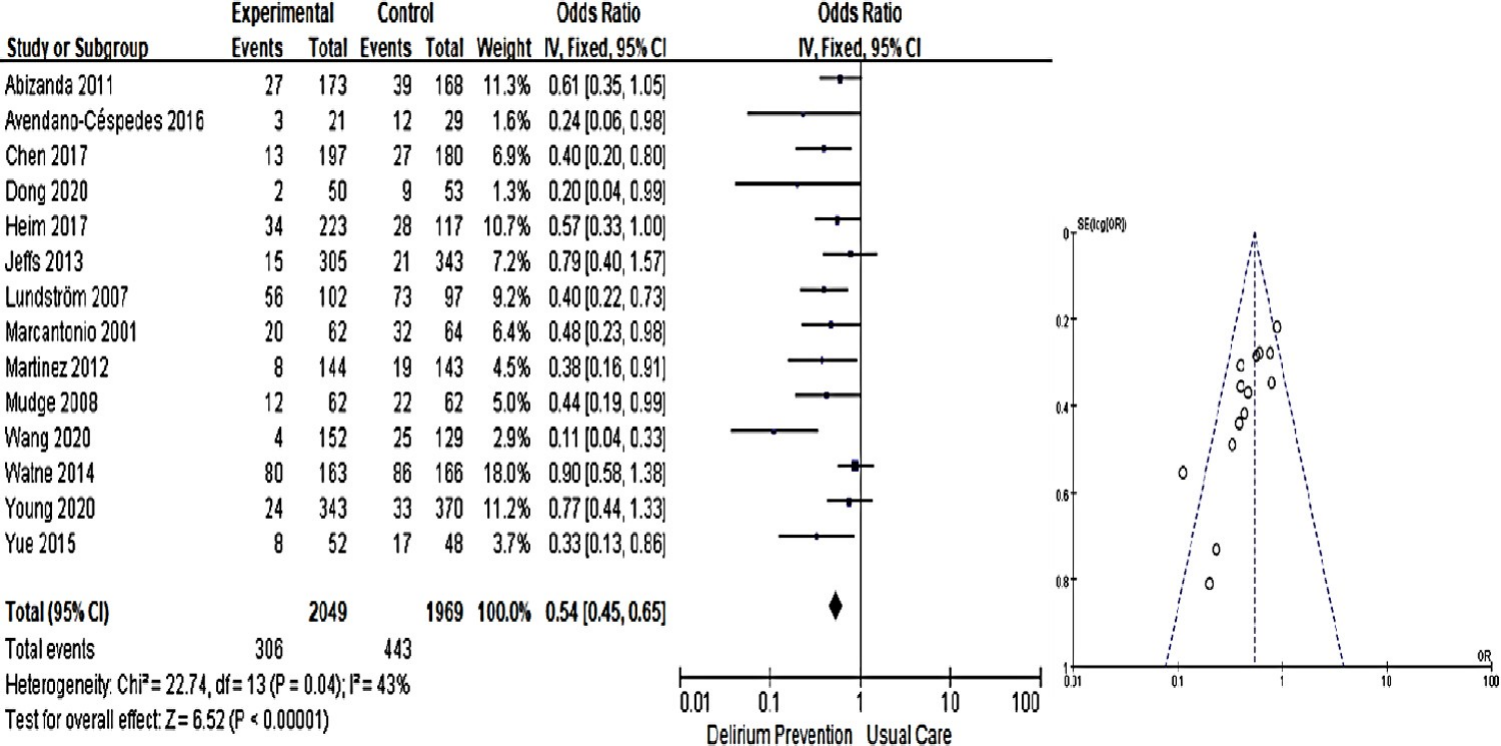
Forest plot of total effect of on the incidence of delirium and funnel plot.

As a result of meta-analysis of 14 studies that reported the effect of non-pharmacological multicomponent intervention as the incidence of delirium, there was a statistically significant difference (OR: 0.54, 95% CI: 0.45~0.65) (Z = 6.52, *p* < 0.001). However, the heterogeneity between studies was intermediate, with I^2^ of 43.0% (X^2^ = 22.74, df = 13, *p* = 0.040) (Fig 3).

#### Effect size on incidence of delirium according to subject group

**Fig 4:**
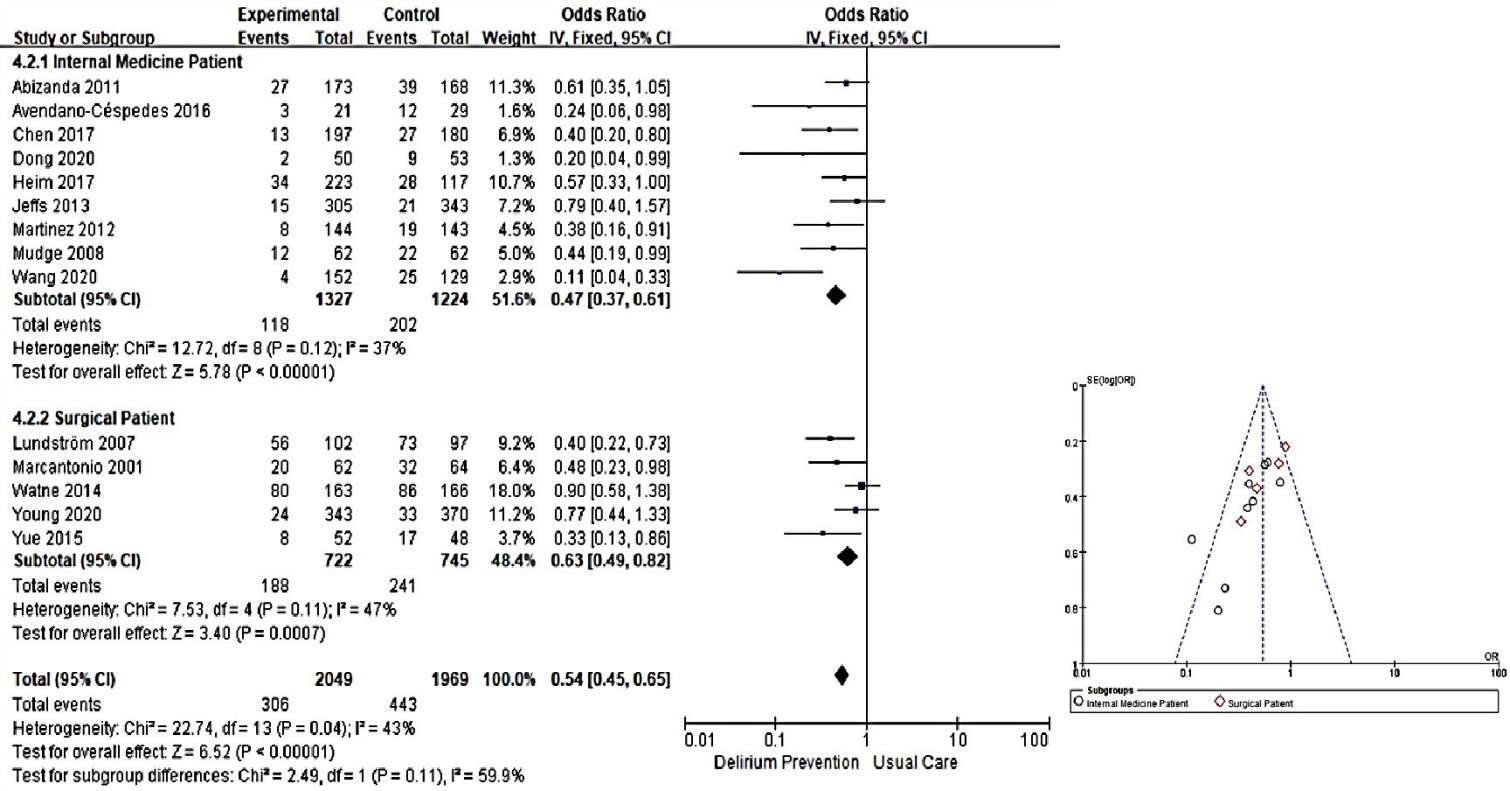
Forest plot of effect of incidence of delirium by subgroup and funnel plot.

Results of meta-analysis of 14 studies that reported the incidence of delirium by dividing them into internal medicine patients and surgical patients according to study subject group are as follows. A total of 9 studies were conducted on patients with internal medical conditions out of a total of 14 studies. As a result of a meta-analysis of results of these 9 studies, there was a statistically significant difference with an OR of 0.47 (95% CI: 0.37~0.61) (Z = 5.78, *p* < 0.001). The heterogeneity between studies was moderate, with I^2^ of 37.0% (X^2^ = 12.72, df = 8, *p* = 0.120). As a result of a meta-analysis of the remaining five studies targeting patients with surgical diseases out of a total of 14 studies, there was a statistically significant difference with an OR of 0.63 (95% CI: 0.49~0.82) (Z = 3.40, *p* < 0.001). The heterogeneity between studies was intermediate with I^2^ of 47.0% (X^2^ = 7.53, df = 4, *p* = 0.110) (Fig 4).

#### Publication bias

Asymmetry was confirmed as a result of confirming the degree of visual symmetry using a funnel plot for the publication bias test. As a result of Egger’s regression test to determine whether the degree of asymmetry was statistically significant, the outcome variable, the delirium incidence rate (t = -3.56, df = 12, *p* < 0.001), was confirmed to have a publication bias. Therefore, as a result of confirming the effect of publication bias on research results using the trim-and-fill method to correct for publication bias, six missing values ​​due to publication bias were estimated. When six papers were used as input and publication bias was corrected, the OR was 0.64, confirming that publication bias affected meta-analysis results because the value changed by more than 10% from an OR value of 0.54 before correcting for publication bias.

## Discussion

This study conducted a systematic review and meta-analysis of non-pharmacological delirium prevention interventions for general ward inpatients conducted in a randomized trial design. Types and components of non-pharmacological delirium prevention interventions were identified and their effects on delirium incidence were confirmed.

A total of 17 studies were included in this study. Of these, 14 studies applied multicomponent intervention and 9 showed an effect in reducing the incidence of delirium. On the other hand, three studies applied single-component intervention and only one of them was effective in reducing the incidence of delirium. Abraha et al. [13] have included 23 multicomponent intervention studies with all experimental design and 10 single-component interventions in a systematic overview study of non-pharmacological delirium prevention interventions in elderly patients. These research trends showed that the direction of non-pharmacological interventions for delirium prevention was being applied as a multicomponent intervention. Since 2012, when Abraha et al. [13] conducted the study, it was confirmed that multicomponent intervention studies designed as RCTs have been continuously conducted. This is because multi-factor interventions can correct or resolve causes of delirium. Such interventions have been established as the main flow of non-pharmacological delirium prevention interventions.

According to Inouye et al. [7], a multicomponent interventional HELP program should be implemented at least 7 days/week, that is, daily. Looking at six studies [A1-A5, A15 in S1 Appendix] that reported the intervention period among the analyzed literature, it was confirmed that the intervention period was at least 5 to 14 days or until discharge. However, many cases, including six studies that reported the intervention period, did not report the number or duration of interventions, or they only reported the number of interventions and the total duration. Therefore, it was difficult to find correlations of reduction in the incidence of delirium with the number and duration of interventions. Hshieh et al. [25] reported an 89% reduction in the risk of developing delirium when the intervention duration and intervention fidelity were high, so the possibility that the intervention period effectively reduces the incidence of delirium cannot be excluded. Therefore, planning the duration of the delirium prevention intervention based on the Inouye et al. [7] will also be a strategy to effectively achieve delirium prevention.

The most included non-pharmacological intervention content is early ambulation. In particular, early ambulation was used in 11 out of 14 multicomponent intervention studies and one single component intervention study. This was almost consistent with the result of early mobilization as the most used content after staff education in a previous study that analyzed non-pharmacological multi-factor interventions applied to elderly patients in the ward [18]. A previous study [26] has reported that white matter blood flow in the frontal and temporal lobes is increased as a result of performing aerobic exercise in elderly patients, which shows that physical activity can improve cognitive ability and prevent delirium.

Mulky et al. [27] have suggested day/night routines, early mobility, reducing sensory impairment, and reorientation are key strategies for evidence-based nursing intervention in delirium prevention. All of the above strategies were applied in 3 literatures, and 2 of them reported that it was effective in reducing the incidence of delirium. It was difficult to conclude that the use of these four strategies could reduce delirium because other contents used in the literature were also effective in reducing the incidence of delirium. However, it is necessary to consider including these four strategies when constructing a delirium prevention intervention. The effectiveness of the four strategies should be confirmed through future research.

In the NICE guideline [15], as the first recommendation for delirium prevention, risk factors for delirium development within 24 hours of hospitalization are assessed. Based on assessment results, a tailored multicomponent intervention tailored to the patient’s individual needs and clinical situation is recommended. As a result of the present meta-analysis, four studies used tailored multicomponent intervention. It was confirmed that some recommendations presented in the NICE guideline [15] were applied. Since risk factors for delirium are different depending on the patient’s situation, it is absolutely necessary to assess risk factors for delirium early when the patient is hospitalized. Based on this, a tailored multicomponent intervention should be constructed and implemented with more extensive use in clinical settings. This is because the patient can receive interventions intensively for areas that need correction so that the burden of work related to the implementation of the delirium prevention intervention can be reduced for medical staff who perform it. In this study, the number of multicomponent intervention contents varied from 2 to 12. The number of contents administered and the effect of reducing the incidence of delirium were inconsistent.

The SIGN guideline [16] suggests ‘prevention, early identification, and treatment of postoperative complications’ as one of its recommendations. This content was included in Part 2 of the literature to be analyzed in this study. It was confirmed that all these were studies of orthopedic patients that included intervention factors reflecting characteristics of surgical patients.

Single component intervention was performed in three studies analyzed in this study. It was confirmed that its effect on delirium incidence rate was inconsistent. A previous study [13] has used bright light, earplug, hydration, music therapy, staff education, reorientation protocol, and Geriatric risk assessment MedGuide software as single component intervention contents for delirium prevention. Among them, only staff education, reorientation protocol, and Geriatric risk assessment MedGuide software were effective in reducing the incidence of delirium. Among the literature analyzed in this study, only the study that implemented music therapy [A11 in S1 Appendix] was effective in reducing the incidence of delirium. The single component intervention has clear limitations in correcting and reducing risk factors for delirium. However, single component intervention is still needed due to the diversity of patient’s individual characteristics and medical institution environment. In the future, it is necessary to accumulate studies to develop interventions for single-component interventions and verify their effectiveness.

In this study, a meta-analysis was performed only on non-pharmacological multi-component intervention studies because contents of single-component intervention were different and the number of studies was small.

As a result of a meta-analysis of the effect of a non-pharmacological multi-component intervention on the incidence of delirium, it was confirmed that such intervention was effective in reducing the incidence of delirium compared to the usual care group. This is similar to the result reported by Siddiqi et al. [9] that a meta-analysis of 7 RCT studies with multicomponent intervention applied to 994 general ward inpatients had a significant effect in reducing the incidence of delirium. However, caution is needed to support the meta-analysis results of Siddiqi et al. [9] because this study excluded cancer patients. In a previous study [9], cancer patients, in addition to the commonly known risk factors for delirium, have been reported to be a factor influencing the occurrence of delirium, unlike patients in other disease groups. Therefore, studies on cancer patients were excluded in the present study. It is necessary to conduct follow-up studies to confirm the effectiveness of non-pharmacological delirium prevention interventions for cancer patients admitted to the general ward.

As a result of sub-analysis, it was confirmed that non-pharmacological multicomponent interventions were effective in reducing the incidence of delirium in both internal and surgical patients. Leon-Salas et al. [2] have performed a meta-analysis for a patient group used in a multicomponent intervention RCT after analyzing four studies with 656 internal medical patients, including cancer patients, and 5 studies with 631 surgical patients. As a result, it was confirmed that multi-component intervention reduced the rate of delirium. Ludolph et al. [18] have reported that non-pharmacological multicomponent intervention is ineffective in a meta-analysis of two studies conducted with 449 internal patients, whereas in 3 studies with 390 surgical patients, non-pharmacologic multicomponent intervention was be effective. Such result is partially consistent with results of the present study. However, since both previous studies [2, 18] reported meta-analysis of cancer patients hospitalized in the ward, there was a limit to directly compare their results with this study.

Finally, as a result of verifying publication bias, asymmetry was confirmed in the Funnel plot. As a result of statistically confirming this result through Egger’s test, it was found that there was a publication bias. As a result of confirming the effect of publication bias with the trim-and-fill method, the RR before the trim-and-fill was increased by about 10% or more. This indicated that the publication bias affected the pooled effect size of this study. When estimating the cause of publication bias through funnel plots, results of the meta-analysis conducted in this study might have overestimated the effect estimates due to the bias caused by failure to publish papers that had no effect on delirium incidence rate. Therefore, it is necessary to be careful when interpreting results.

This study comprehensively and systematically reviewed non-pharmacological delirium prevention interventions for general ward inpatients, performed meta-analyses to determine effects of multicomponent interventions on delirium incidence rates, and presented effects of multicomponent intervention on delirium in general ward inpatients for the first time.

Limitations of this study are as follows. First, many studies included in this meta-analysis did not report the frequency or duration of non-pharmacological interventions. In the case of multicomponent intervention, intervention elements varied from study to study, making it difficult to suggest a standardized strategy for non-pharmacological multicomponent interventions for general ward inpatients. Second, there were only five studies on surgical patients. Since the number of studies was small, careful attention is required when constructing interventions for surgical patients. Third, single-component intervention cannot present an integrated effect estimate through meta-analysis due to different compositions of interventions for each study. Therefore, it was not possible to verify whether single-component intervention had an effect on the actual delirium incidence rate. In the future, it is necessary to present quantitative evidence through follow-up studies to determine the effect of non-pharmacological single-component interventions on delirium in general ward inpatients.

## Conclusions

This study conducted a systematic review of randomized control studies in which non-pharmaceutical delirium prevention interventions were administered to general ward inpatients and performed a meta-analysis to determine effects of interventions on delirium incidence rates.

Non-pharmacologic delirium prevention interventions for general ward inpatients mainly consisted of multicomponent interventions that could reduce delirium risk factors. Intervention content and number/period of interventions were varied according to characteristics of patients and clinical situations for each study. As a result of the meta-analysis, a non-pharmacological multicomponent intervention was effective in reducing the incidence of delirium in each patient group, both internally and externally. Taken together, it was possible to confirm the effectiveness of delirium prevention by applying non-pharmacological interventions, especially multicomponent interventions, for the prevention of delirium in general ward inpatients with a quantitative evidence.

Delirium is one of major causes influencing the clinical prognosis of general ward inpatients. As it has been reported that the use of drugs for the prevention of delirium is not effective, the demand for non-pharmacological interventions for the prevention of delirium is still high. In the future, various non-pharmacological interventional studies targeting ward patients are needed and high-quality studies should be performed to evaluate the effectiveness and scientific basis for decision-making for delirium prevention in general ward inpatients.

## Supporting information

S1 Checklist PRISMA 2020 checklist. (DOCX)

S1 Appendix Appendix: List of included studies. (DOCX)
