# Supplementary material for: Effects of non-pharmacological interventions for preventing delirium in general ward inpatients: A systematic review & meta-analysis of randomized controlled trials
Source: PLoS One. 2022 May 6;17(5):e0268024. doi: 10.1371/journal.pone.0268024 (PMC9075647; doi:10.1371/journal.pone.0268024)
Supplement: S1 Appendix — (DOCX) [file pone.0268024.s002.docx]

**Appendix: List of included studies**

A1. Abizanda P, León M, Domínguez-Martín L, et al. Effects of a short-term occupational therapy intervention in an acute geriatric unit. A randomized clinical trial. *Maturitas*. 2011;69(3):273-278. https://doi.org/10.1016/j.maturitas.2011.04.001

A2. Avendaño-Céspedes A, García-Cantos N, González-Teruel Mdel M, et al. Pilot study of a preventive multicomponent nurse intervention to reduce the incidence and severity of delirium in hospitalized older adults: MID-Nurse-P. *Maturitas*. 2016;86:86-94. https://doi.org/10.1016/j.maturitas.2016.02.002

A3. Chen CC, Li HC, Liang JT, et al. Effect of a modified Hospital Elder Life Program on delirium and length of hospital stay in patients undergoing abdominal surgery: a cluster randomized clinical trial. *JAMA Surg*. 2017;152(9):827-834. https://doi.org/10.1001/jamasurg.2017.1083

A4. Dong Z, Song J, Ge M, et al. Effectiveness of a multidisciplinary comprehensive intervention model based on the Hospital Elderly Life Program to prevent delirium in patients with severe acute pancreatitis. *Ann Palliat Med*. 2020;9(4):2221-2228. https://doi.org/10.21037/apm-20-913

A5. Heim N, Stel H, Ettema R, Mast R, Inouye S, Schuurmans M. HELP! Problems in executing a pragmatic, randomized, stepped wedge trial on the Hospital Elder Life Program to prevent delirium in older patients. *Trials*. 2017;18:220. https://doi.org/I 10.1186/s13063-017-1933-4

A6. Jeffs KJ, Berlowitz DJ, Grant S, et al. An enhanced exercise and cognitive programme does not appear to reduce incident delirium in hospitalised patients: a randomised controlled trial. *BMJ Open*. 2013;3(6):e002569. https://doi.org/10.1136/bmjopen-2013-002569

A7. Lundström M, Olofsson B, Stenvall M, et al. Postoperative delirium in old patients with femoral neck fracture: a randomized intervention study. *Aging Clin Exp Res*. 2007;19(3):178-186. https://doi.org/10.1007/BF03324687

A8. Marcantonio ER, Flacker JM, Wright RJ, Resnick NM. Reducing delirium after hip fracture: a randomized trial. *J Am Geriatr Soc*. 2001;49(5):516-522. https://doi.org/10.1046/j.1532-5415.2001.49108.x

A9. Martinez FT, Tobar C, Beddings CI, Vallejo G, Fuentes P. Preventing delirium in an acute hospital using a non-pharmacological intervention. *Age Ageing*. 2012;41(5):629-634. https://doi.org/10.1093/ageing/afs060

A10. Martínez-Velilla N, Casas-Herrero A, Zambom-Ferraresi F, et al. Effect of Exercise Intervention on Functional Decline in Very Elderly Patients During Acute Hospitalization: A Randomized Clinical Trial. *JAMA Intern Med*. 2019;179(1):28-36. https://doi.org/10.1001/jamainternmed.2018.4869

A11. McCaffrey R, Locsin R. The effect of music listening on acute confusion and delirium in elders undergoing elective hip and knee surgery. *J Clin Nurs*. 2004;13(6B):91-96. https://doi.org/10.1111/j.1365-2702.2004.01048.x

A12. Mudge AM, Giebel AJ, Cutler AJ. Exercising body and mind: an integrated approach to functional independence in hospitalized older people. *J Am Geriatr Soc*. 2008;56(4):630-635. https://doi.org/10.1111/j.1532-5415.2007.01607.x

A13. O'Gara BP, Mueller A, Gasangwa DVI, et al. Prevention of early postoperative decline: a randomized, controlled feasibility trial of perioperative cognitive training. *Anesth Analg*. 2020;130(3):586-595. https://doi.org/10.1213/ANE.0000000000004469

A14. Wang YY, Yue JR, Xie DM, et al. Effect of the tailored, family-involved hospital elder life program on postoperative delirium and function in older adults: a randomized clinical trial. *JAMA Intern Med*. 2020;180(1):17-25. https://doi.org/10.1001/jamainternmed.2019.4446

A15. Watne LO, Torbergsen AC, Conroy S, et al. The effect of a pre- and postoperative orthogeriatric service on cognitive function in patients with hip fracture: randomized controlled trial (Oslo Orthogeriatric Trial). *BMC Med*. 2014;12:63. https://doi.org/10.1186/1741-7015-12-63

A16. Young J, Green J, Farrin A, et al. A multicentre, pragmatic, cluster randomised, controlled feasibility trial of the POD system of care. *Age Ageing*. 2020;49(4):640-647. https://doi.org/10.1093/ageing/afaa044

A17. Yue J. A perioperative multidisciplinary intervention to prevent postoperative delirium in Chinese elderly patients: A cluster randomized controlled pilot study. *The Gerontologist*. 2015;55(Suppl_2):182.
